# Multiple roles of bamboo as a regulator of cyanobacterial bloom in aquatic systems

**DOI:** 10.1038/s41598-022-05506-2

**Published:** 2022-01-31

**Authors:** Aimin Hao, Mengyao Su, Sohei Kobayashi, Min Zhao, Yasushi Iseri

**Affiliations:** 1 College of Life and Environmental Sciences, Wenzhou University, Chashan Academic Town, Ouhai, Wenzhou, 325035 Zhejiang China; 2 National and Local Joint Engineering Research Center of Ecological Treatment Technology for Urban Water Pollution, Wenzhou University, Wenzhou, 325035 China

**Keywords:** Freshwater ecology, Freshwater ecology, Limnology, Biotic

## Abstract

To understand the potential roles of terrestrial bamboo on controlling cyanobacterial blooms in aquatic systems, growth rates of the cyanobacterium *Microcystis aeruginosa* and its competitor algae were examined under different concentrations of bamboo extract. In mono-species cultures with unicellular algal strains, 5.0 g L^−1^ extract treatment suppressed *M. aeruginosa* growth, while it had little effect on the growth of green alga *Scenedesmus obliquus* or promoted the growth of diatom *Nitzschia palea*. In co-species cultures, the extract treatment increased the effect of *S. obliquus* and *N. palea* on the growth of *M. aeruginosa*. Under the extract treatment with a field-collected *M. aeruginosa* population, its cell density declined and its colony was etiolated and sank, while co-cultured *N. palea* increased explosively by invading the colony. These results suggest that bamboo forest stands along banks and artificially installed bamboo poles can affect the aquatic environment for phytoplankton community. Enhancing the growth of competitors, especially diatoms that can invade cyanobacterial colonies, by using extracts or by providing substrates for growth, was suggested to be the major way of the bloom control by bamboo.

## Introduction

Cyanobacterial blooms in aquatic systems, as a consequence of eutrophication caused by human activities, are a major environmental issue worldwide^1–3^. Cyanobacteria produce toxins that are hazardous to both ecosystems and human society^4–6^ and the bloom can eliminate key plants and aerobic animals in the ecosystems by covering the water surface and depletion of oxygen^7–10^. Efforts have been made to reduce nutrient loads from the watershed, and various physical, chemical, and biological control countermeasures have been tested to mitigate cyanobacterial blooms in aquatic systems^3,11^.

Terrestrial plants have roles of modulating the productivity and species composition of adjacent aquatic ecosystems through multiple pathways. Bankside trees limit light availability and algal production while providing leaves for allochthonous production^12–14^, and submerged large woody materials often provide essential habitats for various organisms in aquatic ecosystems^15,16^. Dissolved organic matter (DOM) from terrestrial plants (i.e., leachates and exudates from plant materials), is considered to act as either nutrients^17,18^ or inhibitors for algal growth. Allelopathy of plants has attracted attention for controlling cyanobacterial blooms owing to their selective toxicity and natural degradability^19,20^. Allelopathic effects on cyanobacteria species have been reported for various terrestrial plants^21,22^ as well as aquatic plants^23^.

Bamboo (sub-family Bambusoideae in the family Poaceae) is a widespread and often major forest stand type, especially in Asian countries^24,25^. Bamboo has been planted and utilized since ancient times, and is currently used as food, medicine, and fuel, and as a strong and flexible material for construction and furniture. Bamboo stands are favored for the prevention of soil erosion and for maintaining soil moisture because of its rhizome-root system and the accumulation of litter on the soil surface^25,26^. However, due to rapid growth and reproduction, abandoned bamboo stands have expanded and invaded adjacent forests in recent decades^25,27,28^, which has become a global concern of ecosystem degradation as bamboo invasion can reduce local plant diversity and its relevant ecosystem function^29,30^.

Bamboo can act as either inhibitor or stimulator of growth, depending on others. Bamboo releases allelochemicals that inhibit the growth of other plants in proximity^31,32^. In addition, the antimicrobial activity of bamboo is well known^33^, and leaves and stems have traditionally been used to maintain the freshness of food and water in Asian countries. On the other hand, the nutritional value of bamboo shoots is well known^34^. Bamboo culms contain endophytic lactic acid bacteria and have been used as livestock fodder^35^, and moreover, bamboo is the food of giant panda^36^. A high litter yield in bamboo forests supports the soil microbial community, and microbial diversity sometimes increases after bamboo invasion to other forest types^37–39^. In contrast, the effects of bamboo on biological communities in aquatic systems are not well understood. A few studies have examined the microbial utilization of bamboo litter in streams^40,41^. Despite the limited understanding, bamboo poles and shrubs have traditionally been used for seaweed production and as artificial fish reefs^42^, and they have recently been shown to be suitable substrates in periphyton-based aquaculture systems, which enhance fish production in ponds through periphyton and associated microfauna^43–45^. Notably, bamboo contains high concentrations of silica^46^, which is an essential nutrient for algae, especially diatoms, and bamboo stands accumulate and export downstream a high quantity of silica generated by rock weathering^47,48^.

Stimulating the growth of green algae and diatoms, which are the major competitors of cyanobacteria, may be a key to control cyanobacterial blooms. Certain green algae species grow efficiently and can outcompete cyanobacteria in the absence of elevated CO_2_, temperature, and herbicides^49,50^. Recently, diatom species have been reported to invade and attack cyanobacterial colonies^51,52^, which are a barrier system and vital for bloom formation^53,54^. Recent studies have also investigated the allelopathic effects of periphyton (dominated by green algae and diatoms) on the growth of cyanobacteria^55,56^. Despite such potential beneficial effects of green algae and diatoms on cyanobacteria, few studies have focused on how to stimulate the growth of these competitors to control cyanobacterial blooms.

To understand the potential ability of bamboo to control cyanobacterial blooms, this study examined the effects of bamboo extract on the growth of the widespread cyanobacterium *Microcystis aeruginosa* and its major competitors, green alga *Scenedesmus obliquus*, and diatom *Nitzschia palea*. The growth of the three algal species was examined by using bamboo extracts of different concentrations in mono- and co-species cultures. We hypothesized that the extract suppresses the growth of *M. aeruginosa*, while it promotes or has no effect on the growth of *S. obliquus* and *N. palea*, and that the effect of the competitors on *M. aeruginosa* increased with the extract. Although a standard strain of unicellular *M. aeruginosa* was used for the basic experiments, colony-forming *M. aeruginosa* collected from a field lake was also used to understand how algal responses to the extract vary with the barrier system of cyanobacteria.

## Results

### Algal growth in mono-species culture

**Figure 1:**
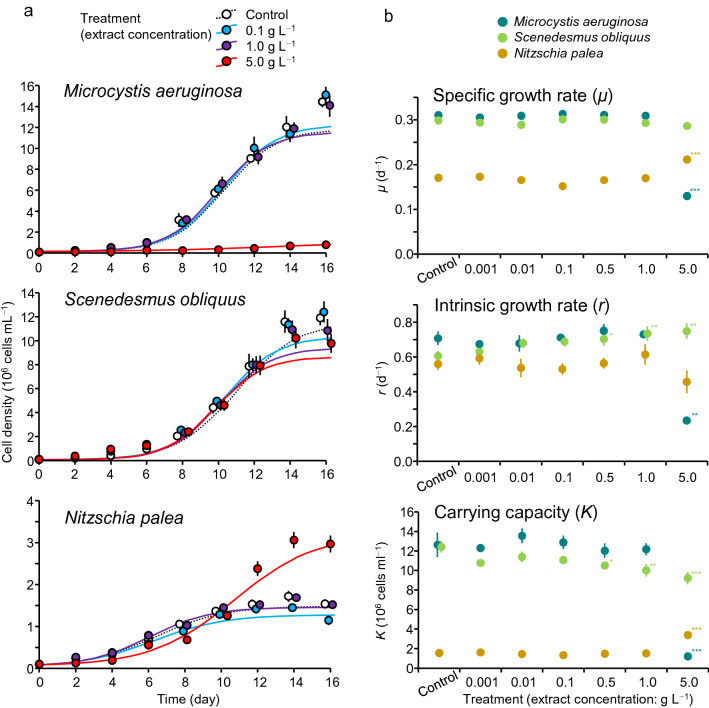
Growth pattern (**a**) and growth parameters (**b**) of each algal species in different treatments in the mono-culture system (the control and selected three treatments are shown in **a**). Each growth curve was fitted with a logistic model. Error bars are SD (*n* = 3). Asterisks denote a significant difference between the control and extract treatments (Dunnett’s test, ^*^: *p* < 0.05, ^**^: *p* < 0.01, ^***^: *p* < 0.001).

The three algal species were incubated in cultures with different bamboo extract concentrations (bamboo: *Semiarundinaria densiflora*) under a constant temperature and light cycle condition for 16 days, and the growth was monitored by counting algal cells every 2 days (Fig. 1a). For each culture, the growth curve was fitted with a logistic function, and growth parameters (*μ*: specific growth rate, *r*: intrinsic growth rate, and *K*: carrying capacity) were determined (Fig. 1b). In some cases, the logistic curve could not accurately follow the actual growth, and *K* may be underestimated (e.g., *M. aeruginosa* in some treatments, Fig. 1a).

The three algal species responded differently to the bamboo extract treatments. The growth of *M. aeruginosa*, in terms of *μ*, *r*, and *K*, differed little between the control and extract treatments up to 1 g L^−1^, whereas it was significantly lower in the 5 g L^−1^ treatment than in the control (*t* = − 54.7, *p* < 0.001) (Fig. 1b). The cell density at the end of the experiment in the 5 g L^−1^ treatment was one-eighteenth of that in the control. The *μ* of *S. obliquus* differed little between the control and each extract treatment, whereas *r* tended to increase (by 23.6% from the control to 5 g L^−1^) and *K* tended to decrease (by 25.7% from the control to 5 g L^−1^) with extract concentration. This reflects an increase in initial growth while a decrease in final cell density with extract concentration. In contrast, *μ* and *K* of *N. palea* differed little between the control and extract treatments up to 1 g L^−1^ concentration, while they were significantly higher in the 5 g L^−1^ treatment than in the control (*t*: 10.33 and 22.38, *p* < 0.001 and < 0.001, respectively). The cell density in the 5 g L^−1^ treatment initially increased relatively slowly, but it was almost double that of the control at the end (Fig. 1a).

**Figure 2:**
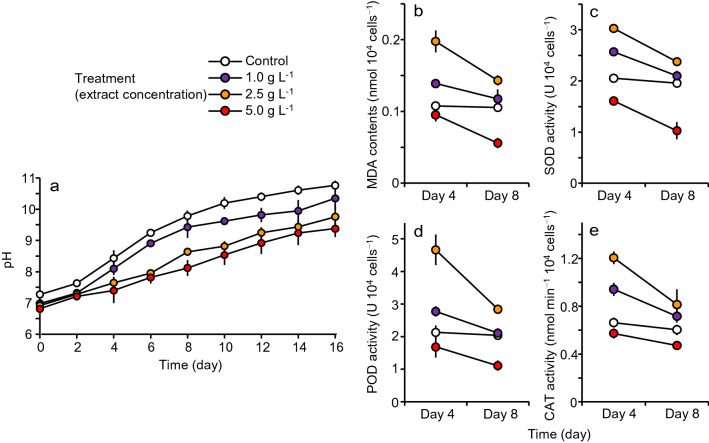
Changes in pH (**a**), malondialdehyde (MDA) content (**b**), superoxide dismutase (SOD) activity (**c**), peroxidase (POD) activity (**d**), and catalase (CAT) activity (**e**) in *Microcystis aeruginosa* mono-species culture. Error bars are SD (*n* = 3).

The pH of culture, lipid peroxidation (malondialdehyde: MDA), and activities of enzymes (superoxide dismutase: SOD, peroxidase: POD, catalase: CAT) were measured as physiological status of *M. aeruginosa* in the control and three extract treatments (1.0, 2.5, 5.0 g L^−1^). The pH increased with time, and it was consistently higher in the control than in the extract treatments and higher in the 1 g L^−1^ treatment than in the 2.5 and 5.0 g L^−1^ treatments after day 4 (Fig. 2a). The MDA content and enzyme activities (SOD, POD, and CAT) decreased from day 4 to 8 in the extract treatments (Fig. 2b–e). On each day, they were highest in the 2.5 g L^−1^, followed by the 1.0 g L^−1^ and the control, and they were lowest in the 5.0 g L^−1^.

### Algal growth in co-species culture

**Figure 3:**
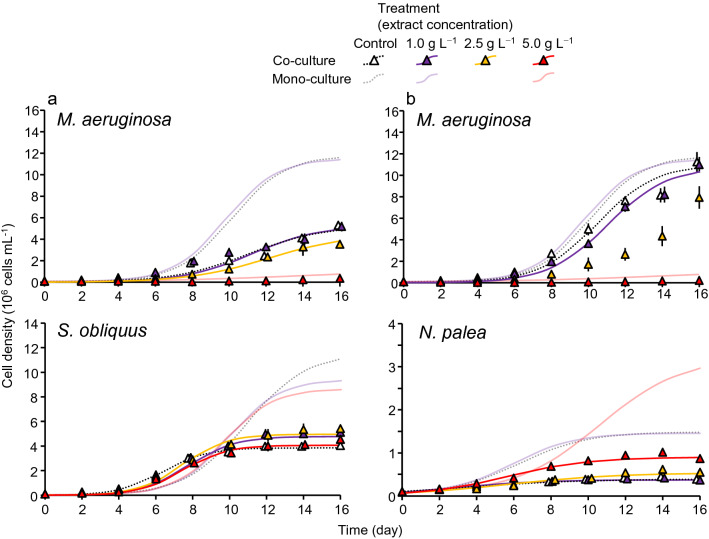
Growth pattern of algal species in different treatments in co-culture systems (**a**: *Microcystis aeruginosa* and *Scenedesmus obliquus*, **b**: *M. aeruginosa* and *Nitzschia palea*). Growth curves were fitted with logistic models except for some cases of *M. aeruginosa*. Error bars are SD (*n* = 3). Growth curves of each species in the mono-culture (Fig. 2) are shown for comparison.

*M. aeruginosa* and either *S. obliquus* or *N. palea* were co-cultured in the control and extract treatments (1.0, 2.5, 5.0 g L^−1^) under the same condition with the mono-culture system, and the growth was monitored for each species (Fig. 3a, b).

**Figure 4:**
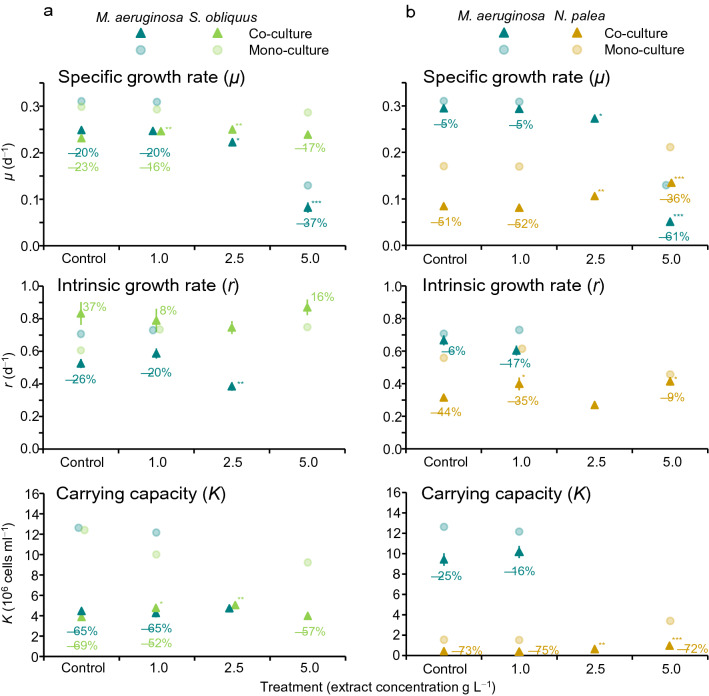
Growth parameters (*μ*, *r*, *K*) calculated for each species in different treatments in co-culture systems (a: *Microcystis aeruginosa* and *Scenedesmus obliquus*, b: *M. aeruginosa* and *Nitzschia palea*). Error bars are SD (*n* = 3). Asterisks denote a significant difference between the control and extract treatments for each species (Dunnett’s test, ^*^: *p* < 0.05, ^**^: *p* < 0.01, ^***^: *p* < 0.001). Growth parameters of each species in the mono-culture and percentage decrease from mono- to co-culture systems for each parameter are also shown. Values of *r* and *K* were not obtained for *M. aeruginosa* in some cases.

In the *M. aeruginosa* and *S. obliquus* co-culture system (Figs. 3a and 4a), *μ* of *M. aeruginosa* was significantly lower in the 5 g L^−1^ treatment than in the control (*t* = − 22.75, *p* < 0.001). The values of *r* and *K* were not obtained at 5 g L^−1^ due to a non-sigmoidal change in cell density. The *μ* and *r* in the 2.5 g L^−1^ treatment were also significantly lower than those in the control (*t*: − 3.53 and − 4.91, *p* = 0.019, and 0.005, respectively). Growth was lower in the co-culture system compared to the mono-culture system (Figs. 3a and 4a); the percentage decrease in *μ* from mono- to co-culture was greater for the 5 g L^−1^ (− 37%) than for the control (− 20%).

In the same co-culture system, *μ* and *K* of *S. obliquus* were significantly greater in the 1.0 g L^−1^, (*t*: 4.31 and 3.30, *p*: 0.006 and 0.027, respectively) and 2.5 g L^−1^ (*t*: 5.40 and 4.16, *p*: 0.002 and 0.008) than in the control (Fig. 4a). The values of *μ* and *K* decreased while *r* increased from the mono- to co-culture system. The percentage decreases in *μ* and *K* from mono- to co-culture were small for the 5 g L^−1^ (− 17% and − 57%, respectively) than the control (− 23% and − 69%).

In the *M. aeruginosa* and *N. palea* co-culture system (Figs. 3b and 4b), *μ* of *M. aeruginosa* was significantly and substantially lower in the 5 g L^−1^ treatment than in the control (*t* = − 42.24, *p* < 0.001). The *μ* was also significantly lower in the 2.5 g L^−1^ treatment than in the control (*t* = − 3.83, *p* = 0.012). Values of *r* and *K* were not obtained in the 2.5 and 5 g L^−1^. Growth was lower in the co-culture system compared to the mono-culture system (Figs. 3b and 4b); the percentage decrease in *μ* from mono- to co-culture was greater in the 5 g L^−1^ treatment (− 61%) than in the control (− 5%).

In the same co-culture system, *μ* and *K* of *N. palea* were significantly higher in the 2.5 g L^−1^ (*t*: 4.11 and 4.53, *p*: 0.008 and 0.005, respectively) and 5.0 g L^−1^ (*t*: 9.63 and 12.21, *p*: < 0.001 for both) treatments than in the control, and *r* was significantly higher in the 1.0 g L^−1^ (*t* = 3.37, *p* = 0.024) and 5.0 g L^−1^ (*t* = 3.97, *p* = 0.010) treatments than in the control (Fig. 4b). The percentage decrease in *μ* and *r* from mono- to co-culture was smaller in the 5 g L^−1^ (− 44% and − 9%, respectively) than in the control (− 51% and − 44%).

### Growth in co-species culture with colony-forming *M. aeruginosa*

**Figure 5:**
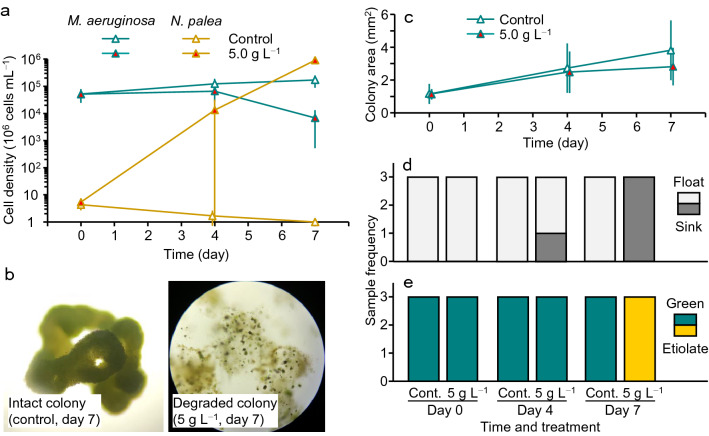
Growth patterns of *Microcystis aeruginosa* and *Nitzschia palea* (**a**) and changes in colony of *M. aeruginosa* (**b**), including size (**c**), float/sink status (**d**), and color state (**e**), in the control and 5 g L^−1^ treatment in co-culture with colony-forming *M. aeruginosa*. Error bars are SD (*n* = 3).

Field collected colony-forming *M. aeruginosa* and *N. palea* were co-cultured in the control and 5 g L^−1^ extract treatment (Fig. 5a); differences in growth between the control and treatment were more conspicuous as compared to the co-culture with singular cells (Fig. 3b). In the control, *M. aeruginosa* increased more than threefold during the 7-day experiment (*μ*: 0.17 d^−1^), whereas *N. palea* decreased to less than one-fourth (Fig. 5a). In contrast, in the 5.0 g L^−1^ treatment, *M. aeruginosa* decreased to less than one-seventh during the experiment, whereas *N. palea* increased to more than 10^5^-fold during the experiment (*μ*: 1.73 d^−1^). Such an explosive increase was not observed in any species or treatments in mono-culture nor in co-culture systems with single cells.

Differences between the control and treatment in the state of *M. aeruginosa* colonies were also evident (Fig. 5b). Although the colony size increased in both the control and the 5 g L^−1^ treatments (Fig. 5c), most colonies in the 5 g L^−1^ treatment were degraded and fragmented at the end of the experiment. In addition, initially intact and floating colonies all sank to the bottom with etiolation in the 5 g L^−1^ treatment by day 7 (Fig. 5d, e). Colonies were at the bottom since day 4 in one of the 5 g L^−1^ triplicates (Fig. 5d), in which *N. palea* started increasing from day 4 (a large variation among triplicates is shown in Fig. 5a).

## Discussion

This study demonstrated the effects of bamboo extract on the growth of three algal species based on batch culture experiments. Both mono- and co-culture experiments suggest that the extract can suppress the growth of the cyanobacterium *M. aeruginosa*, while it can promote the growth of the competitive diatom *N. palea*. Multiple pathways of the effect of bamboo on cyanobacteria are possible, including direct and indirect effects of the extract. Few studies have shown such opposing effects of extracts between cyanobacteria and other algal species; thus, these observations might be specific to bamboo and related plants. The remarkable effect of bamboo extract on colony-forming cyanobacteria suggests that bamboo may perform advantageously in field ponds and lakes where colony-forming cyanobacteria occur, as allelopathic effects of other plants are usually tested for singular cells without protecting systems, which rarely occur in the stages of proliferation.

We revealed that the growth of *M. aeruginosa* can be largely inhibited by bamboo extract at a concentration of 5 g L^−1^. Although the effect on growth was less clear in 1.0 and 2.5 g L^−1^ treatments, physiological changes induced by these treatments were suggested by the culture pH and stress indicators. An increase in pH to > 10 in cultures is normal for *M. aeruginosa*, which is adapted to higher pH and the availability of CO_2_ in water^59^. Lower pH in the extract treatments than in the control suggests a decline in algal photosynthetic activity by the extract. The stress indicators MDA content and activities of antioxidant enzymes (SOD, POD, and CAT) behaved in a concentration-dependent manner, except for the 5.0 g L^−1^ treatment. The minimum levels of these indicators in the 5.0 g L^−1^ treatment could suggest a malfunction of cells rather than a reduced stress in the treatment.

In this study, extracts of 1 g L^−1^ and lower concentrations had little or no effect on the growth of *M. aeruginosa*. Although previous studies using other plants often showed effects of extracts with concentrations of 1–10 g L^−1^ or higher [e.g.,^58,60^], effects with much lower concentrations have also been reported (e.g., 0.2 g L^−1^ of leaves of *Eucalyptus* trees^61^; 0.05 g L^−1^ of decomposed barley straw^62^). Thus, the algistatic level of bamboo extract in this study was not specifically high among reported effective plants.

No apparent decrease in the growth of *S. obliquus* and *N. palea*, even in the 5 g L^−1^ treatment, suggests that the bamboo extract can selectively inhibit the growth of cyanobacteria. Selective inhibitory effects of plant extracts on cyanobacteria have been demonstrated in other studies^63,64^, which could be attributed to the lack of a defense system in cyanobacteria against allelochemicals such as those developed in green algae and diatoms. Our results rather showed a promotive effect of bamboo extract on the growth *of N. palea* in the 5 g L^−1^ treatment. Few studies have shown such opposing effects between cyanobacteria and other algal species simultaneously. Eladel et al.^65^ reported that extract of rice straw inhibited the growth of cyanobacteria (*Anabaena*), while stimulating the growth of green algae (*Chlorella*). According to their review, extracts from barley and rice straws often inhibit the growth of cyanobacteria, including *Microcystis*, while stimulating the growth of green algae and diatoms as a result of high nutrient (NO_3_ and PO_4_) release^65^. It is interesting that both straw plants and bamboo belong to the family Poaceae and have many common properties, including high silica content^46^.

It appears that, in the co-culture systems without bamboo extract, *M. aeruginosa* and *S. obliquus* affected each other with less competitive advantage, whereas *M. aeruginosa* was competitively superior to *N. palea*. Growth parameters of each species were lower in the co-culture system than in the mono-culture system, except for the *r* of *S. obliquus*. It is known that *S. obliquus* can outcompete *M. aeruginosa* in cultures without herbicides or with low to moderate pH and temperature conditions^49,66^. The diatom *N. palea* has been shown to be a poor competitor for nutrients among various algal species^67^.

The percent change in growth parameters from the mono-culture to co-culture suggests that the relationship between *M. aeruginosa* and its competitor was modified by the bamboo extract. A greater percentage decrease in the *μ* of *M. aeruginosa* in the 5.0 g L^−1^ treatment than in the control implies that negative impacts from competitors increased in the presence of the extract. Impaired growth of *M. aeruginosa* in co-culture was also observed as a non-sigmoidal increase (e.g., decrease, slow exponential increase) of cells in the 2.5 and 5.0 g L^−1^ treatments. In contrast, a smaller percentage decrease in *μ* of *S. obliquus* and *N. palea* for the 5.0 g L^−1^ treatment compared to the control implies that the negative impact by *M. aeruginosa* was reduced by the extract. The numerical superiority between *M. aeruginosa* and *N. palea* in the control was completely reversed in the 5.0 g L^−1^ treatment. The greater effects of *N. palea* on *M. aeruginosa* in the 5.0 g L^−1^ treatment compared to those of *S. obliquus* may be attributed to its periphytic nature, as discussed in more detail in the following paragraph.

The ability of bamboo extract to control *M. aeruginosa* by promoting the growth of competitors was enhanced in the colony-forming *M. aeruginosa*. In co-culture with colony-forming *M. aeruginosa*, *M. aeruginosa* increased and *N. palea* decreased in the control, whereas in the 5 g L^−1^ treatment, *M. aeruginosa* decreased by less than one-seventh and *N. palea* increased more than 10^5^-fold. It appears that the colony triggered a rapid death of *M. aeruginosa*, rapid growth of *N. palea*, or both in the bamboo extract. A unique feature of *N. palea* is its adhesion to, and gliding movement on, the substrate, which enables them to invade and consume the colony of *M. aeruginosa*^51,52^. We observed that the colonies of *M. aeruginosa*, which were initially intact and floating, became partially fragmented, etiolated, and sank (settled to the bottom) in the extract treatment. The invasion of *M. aeruginosa* by *N. palea* was possibly accelerated by the settlement of the colony. However, the process that induced the loss of floating ability of the colonies, whether they were degraded by the extract or by the invasion of *N. palea*, is unknown.

**Figure 6:**
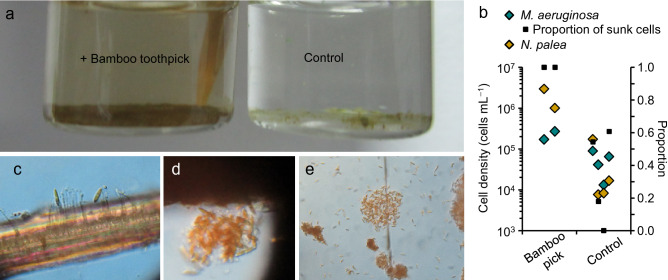
Algal responses to bamboo toothpick in culture (**a**: experiment culture, **b**: cell density of *Microcystis aeruginosa* and *Nitzschia palea* with the proportion of sunk cells for *M. aeruginosa*, **c**–**e**: microscopic observation of *N. palea* attached on bamboo fragments individually and as a flock.

Our supplemental observations show the importance of bamboo as a substrate for the growth of *N. palea* (Fig. 6). The colony-forming *M. aeruginosa* with *N. palea* taken from Lake Taihu was inoculated equally into six vials (8 mL) with BG-11 medium, and a commercial toothpick made of bamboo was placed in two of the six vials. Incubation conditions were the same as those in the main experiments. There was no visible change in the color of the cultures for several weeks, but the color in vials with toothpicks changed to yellowish after 7–8 weeks (Fig. 6a). Microscopic observations at day 60 revealed that in the vials with toothpicks all *M. aeruginosa* had sunk and *N. palea* had increased substantially (Fig. 6b). In addition, a large number of *N. palea* formed flocks and adhered to the surface of the toothpicks (Fig. 6c–e). The yellowish color was primarily *N. palea* that adhered to the vial walls. The lack of coloring of the culture together with a lack of decrease in total number (float + sink) of *M. aeruginosa* suggest that the leachate from the toothpicks was limited. This implies that the toothpick contributed as a growing substrate for *N. palea* and subsequently as a source of invaders for the colony of *M. aeruginosa*. The increase of *N. palea* by bamboo addition was observed in another mono-culture experiment using different bamboo sticks, while such response was not found for woody and plastic sticks (personal observations, MS).

Our results are still insufficient to generalize impacts of bamboo on algal species in fields. We used the extract of bamboo culms collected from a certain bamboo stand. Our supplemental observation using the commercial bamboo toothpicks (Fig. 6) partially supported the findings in the extract experiments. Further studies using bamboos from different stands, different organs (e.g., leaves, roots), different ages, and different bamboo species are required to understand general impacts of bamboo. Furthermore, the impacts of bamboo may differ between sterilized and unsterilized extracts. Although we autoclaved to sterilize the extract, some compounds might have been destroyed by this process. In addition, fungi and bacterial activities may be high on unsterilized extract or natural litter of bamboo^35,36^. In another observation, colony-forming *M. aeruginosa* in vials without *N. palea* disappeared within a few days after adding an unsterilized bamboo culm piece (collected from different stands) in the vials (personal observations, SK). Both leachates from the bamboo and microbial activities associated with the bamboo are possible causes of the disappearance.

**Figure 7:**
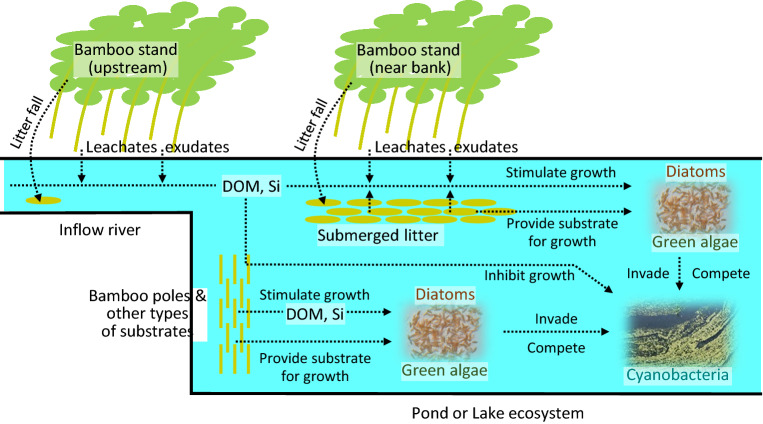
Possible pathways of bamboo control on cyanobacteria. DOM: dissolved organic matter, Si: silica.

We suggest that bamboo can suppress *M. aeruginosa* through inhibiting its growth by dissolved chemicals and through stimulating the growth of competitors, especially diatoms, either as a result of dissolved chemicals or of providing plant material as a substrate for growth (Fig. 7). The effective extract concentration in this study (i.e., 1–5 g L^−1^) is not easy to obtain in the field other than in small ponds, because 1 and 5 kg of bamboo is needed for 1 m^3^ of water. Planting bamboo stands along the shoreline would increase DOM associated with bamboo and also increase the available substrates for diatoms and green algae (Fig. 7). In addition, bamboo poles are known to be effective substrates for developing periphyton biofilms dominated by green algae and diatoms^43–45^. Periphyton has also garnered the attention of researchers for controlling the growth of cyanobacteria^55,56^. Bamboo enables such synergetic effects of DOM and substrates in controlling cyanobacteria, which would be more feasible for application in the field.

The colony of *M. aeruginosa*, which plays a key role in dominance and bloom formation, may also be a clue to degeneration. The colony (sheath and mucilage), which mainly consists of extracellular polysaccharides, protects cells from predators, and mitigates stress under less suitable environmental conditions^54,59^. The colony also provides nutrients for their cells under low nutrient conditions and it harbors a symbiotic bacterial community^68,69^. However, the results of the present and previous studies^52^ suggest that the colony can turn into a substrate for attachment and food for certain algal species. It is assumed that the barrier system of the colony is weakened by a certain stress from the external environment (e.g., in the presence of bamboo extract), which subsequently allows the invasion of competitors.

The control of cyanobacteria using bamboo might be categorized as a biological control in a conventional classification. However, using bamboo differs from common case, where a plant or animal that directly suppresses cyanobacteria, is introduced and maintained in an aquatic system. Using bamboo also differs from cases that focus on allelochemicals of plants, in which selectivity, algistatic level, dosage, and persistence, are major concerns, more similar to chemical control. Because we expect that modulating the water- and habitat-environment to enhance competitors of cyanobacteria is the major way that bamboo effects are mediated, interventions using bamboo could be termed habitat-environmental control. This study revealed the potential roles of bamboo extract in controlling cyanobacteria using widespread species; however, several important questions, including relevant chemicals, variations among plant organs and among life stages, the processes involved in the increase and decrease of algal species, and the actual effects in the field need to be addressed through further studies.

## Methods

### Material preparation

Three common freshwater phytoplankton species, cyanobacterium *Microcystis aeruginosa*, green alga *Scenedesmus obliquus*, and diatom *Nitzschia palea*, were used. Unicellular *M. aeruginosa* (FACHB-912), *S. obliquus* (FACHB-14), and *N. palea* (FACHB-2263) were obtained from the Freshwater Algae Culture Collection at the Institute of Hydrobiology, Chinese Academy of Science, China. In addition, colony-forming *M. aeruginosa* was isolated from natural populations in Lake Taihu, Zhejiang Province, China, in October 2020. Medium BG-11 and CS1 (Supplementary Table S1) were purchased from the same institute. Medium BG-11 was used to culture *M. aeruginosa*, and *S. obliquus* and CS1 was used to culture *N. palea*.

A 15 mL liquid of each algal species was transferred to a sterile Erlenmeyer flask, in which cells were acclimated for three days under a 12:12 h light/dark cycle and 1000 lx light intensity at 25 °C. The liquid was then transferred to another sterile flask containing 30 mL of the culture medium. The cells were cultivated under a 12:12 h light/dark cycle and 2000 lx light intensity at 25 °C. Algal cells in the exponential growth phase were used for the experiments.

Colony-forming *M. aeruginosa* collected in a sterile bottle from Lake Taihu was transported in a cooler box to the laboratory. A 10 mL was sampled from the upper layer of floating algal materials (mainly *M. aeruginosa*) and added to 120 mL of BG-11 medium in a sterile flask. The culture was incubated under a 12:12 h light/dark cycle and 2000 lx light intensity at 25 °C for a several days until *M. aeruginosa* entered the growing phase.

An indigenous and widespread bamboo (short-spike bamboo, *Semiarundinaria densiflora* (Rendle) T.H. Wen) was collected in a public place in Wenzhou, Zhejiang Province, China. The collection of the wild plants and the following processes were done in accordance with rules of plant collection in public places in China. The bamboo species was identified by Yonghua Zhang (College of Life and Environmental Sciences, Wenzhou University) and the voucher specimen was deposited in the Herbarium of Wenzhou University (WZU) (collection number: HAM2021001). Several culms of fresh and presumably young ramets (height: 2–3 m) were collected at the edge of a bamboo stand in summer. Peeled pieces from various positions of the culms were rinsed with ultrapure water and dried in a shaded and ventilated place indoors (Supplementary Fig. S1). The dried pieces were then cut and ground using a mortar and pestle. A 10 g of bamboo powder was mixed with 200 mL ultrapure water in a flask to obtain a 50 g L^−1^ concentration. The flask containing the aqueous solution was autoclaved at 120 °C for 30 min to obtain the bamboo extract. After cooling, the extract was transferred to a sterile tube and centrifuged at 10,000 × *g* for 10 min at 25 °C. The supernatant was divided into several aliquots and stored at − 20 °C.

In the following experiments, different amounts (0.02–10 mL) of the bamboo extract were added to the media adjusted to 100 mL in sterile flasks with cotton plugs to give different extract concentrations (0.001–5.0 g L^−1^).

### Mono-culture experiments

Control and bamboo extract-treated flasks (extract concentrations: 0.001, 0.01, 0.1, 0.5, 1.0, 5.0 g L^−1^) were prepared for each algal species using BG-11 for *M. aeruginosa* and *S. obliquus* and the CSi for *N. palea* (Supplementary Fig. S1). Each 250 mL flask was inoculated with an algal species with an initial cell density of *ca.* 1.0 × 10^5^ cells mL^−1^. All six treatments were performed in triplicate for each species. All the flasks were placed in a 25 °C incubator with 12:12 h light/dark cycle and 3000 lx illumination, and they were shaken, and positions randomly rearranged, three times per day. The experiment was performed for 16 days, and a 30 μL subsample was removed from each flask every two days. The cell density in each subsample was determined by counting the cells three times (with each time using 10 μL) using a hemocytometer under a microscope. To homogenize algal cells in culture, pipetting to flash algal cells adhered on the flask wall and shaking were appropriately done before the subsampling. Because *S. obliquus* formed four-celled coenobia, we counted the number of coenobia.

To measure the physiological status of *M. aeruginosa*, additional control and bamboo extract flasks (extract concentrations: 1.0, 2.5, 5.0 g L^−1^) were prepared using BG-11. Each flask was inoculated with *M. aeruginosa* with an initial cell density of *ca.* 1.0 × 10^5^ cells mL^−1^. All four treatments were performed in triplicate, with incubation as previously described. As an indicator of physiology, the pH of the culture was measured using a portable water quality meter (HQ40D, Hach, Loveland, USA) every two days. A 10 mL subsample was removed from each flask on days 4 and 8 for the physiological measurements.

### Co-culture experiments

Control and bamboo extract-treated flasks (extract concentrations: 1.0, 2.5, 5.0 g L^−1^) were prepared for two co-culture systems (*M. aeruginosa* vs. *S. obliquus* and *M. aeruginosa* vs. *N. palea*) using BG-11 medium modified by adding 100 mg L^−1^ Na_2_SiO_3_·9H_2_O to the original (Supplementary Fig. S1). Each 250 mL flask was inoculated with two algal species with an initial cell density of *ca.* 1.0 × 10^5^ cells mL^−1^ for each species. All treatments were performed in triplicate for each system. Cultivation conditions and sampling procedures were identical to those used in the mono-culture experiments.

### Co-culture experiments using colony-forming *M. aeruginosa*

Control and bamboo extract-treated wells were prepared on a sterile culture plate using 2 mL modified BG-11 medium, and extract concentration of 5.0 g L^−1^ (Supplementary Fig. S1). An intact colony of *M. aeruginosa* was isolated under a microscope and inoculated into each well. The initial density of *N. palea*, which was originally attached to the colony of *M. aeruginosa*, was not controlled, resulting in 2–8 × 10^4^ cells mL^−1^. Control and extract treatments were performed in triplicate. The cultures were incubated under a 12:12 h light/dark cycle and 3000 lx light intensity at 25 °C, and the plate was shaken once every day until day 7. A 10 μL subsample was removed from each well on days 4 and 7 for cell counting of *N. palea*. The colony of *M. aeruginosa* was partially removed for cell counting and the cell number for the whole colony was estimated based on the subsampled area.

### Growth calculation

The specific growth rate (*μ*, d^−1^) of each species was calculated based on the initial and final cell densities using the following equation:

$$\mu =\frac{ln{C}_{2}-ln{C}_{1}}{{t}_{2}-{t}_{2}}$$

where *C*_1_ and *C*_2_ are the cell densities (cells mL^−1^) at time (d) *t*_1_ and *t*_2_, respectively. In this study, *t*_1_ was 0, and *t*_2_ was 16 for the mono- and co-culture experiments, whereas *t*_2_ was 7 for the experiment using the colony-forming *M. aeruginosa*.

The intrinsic growth rate (*r*), which represents the initial growth rate, and carrying capacity (*K*), which represents the final equilibrium density, were also calculated by fitting a growth curve with the following logistic function:

$$N(t)=\frac{K}{1+(K-{N}_{0}){e}^{-rt}/{N}_{0}}$$

where *N*_0_ is the initial cell density (cells mL^−1^), *N*_t_ is the cell density (cells mL^−1^) at time *t*, *K* is the carrying capacity (cells mL^−1^), and *r* is the intrinsic growth rate (d^−1^). First, *r* and *K* were calculated by solving the y-intercept and x-intercept, respectively, in a linear regression of per-capita growth rate (*dN N*_*t*_^−1^ *dt*^−1^) by cell density (*N*_*t*_)^57^ (Supplementary Fig. S2). Then, *N*_0_ was estimated by non-linear regression starting with *N*_0_ of 10 using the nlsm function in R (version 4.0.3; R Development Core Team, Vienna, Austria).

### Oxidative compounds and enzyme activities

Lipid peroxidation, malondialdehyde (MDA), and antioxidant enzymes have been used as indices of oxidative damage to *M. aeruginosa*^58^. The MDA content and activities of superoxide dismutase (SOD), peroxidase (POD), and catalase (CAT) in the cells were measured using assay kits (SOD-2-Y, POD-2-Y, CAT-2-W, MDA-2-Y, Suzhou Keming Biotechnology Co., Ltd., Suzhou, China) (Supplementary Table S2).

### Statistical analysis

Dunnett’s test, which compares extract treatments (6 and 3 treatments for mono- and co-culture, respectively) with the control, was done for *μ*, *r*, and *K* of each species (*n* = 3 for each treatment) using R with the package of “multcomp.” An *α* value of 0.05 was used to determine the significance of the effects.

## Supplementary Information

Supplementary Information.

## Data Availability

The data supporting the findings of this study are available within the article and https://figshare.com/s/2c76b602a13cb4a140c3.
