# Supplementary material for: Multiple roles of bamboo as a regulator of cyanobacterial bloom in aquatic systems
Source: Sci Rep. 2022 Jan 31;12:1605. doi: 10.1038/s41598-022-05506-2 (PMC8803990; doi:10.1038/s41598-022-05506-2)
Supplement: Supplementary file 1 — Supplementary Information. [file 41598_2022_5506_MOESM1_ESM.docx]

Supplementary Materials for

**Multiple roles of bamboo as a regulator of cyanobacterial bloom in aquatic systems**

Aimin Hao, Mengyao Su, Sohei Kobayashi, Min Zhao, Yasushi Iseri

Corresponding Author:

Sohei Kobayashi (koba@wzu.edu.cn), Yasushi Iseri (iseri@wzu.edu.cn)

**Table S1**. Ingredients in BG-11 and CSi media.

| BG-11 | |  | CSi | |
| --- | --- | --- | --- | --- |
| Ingredient | Concentration |  | Ingredient | Concentration |
| NaNO_3_ | 1.5 g L^−1^ |  | Ca(NO_3_) ·4H_2_O | 0.15 g L^−1^ |
| K_2_HPO_4_ | 0.04 g L^−1^ |  | KNO_3_ | 0.1 g L^−1^ |
| MgSO_4_·7H_2_O | 0.075 g L^−1^ |  | MgSO_4_·7H_2_O | 0.04 g L^−1^ |
| CaCl_2_·2H_2_O | 0.036 g L^−1^ |  | β-Na_2_ glycerophosphate·5H_2_O | 0.025 g L^−1^ |
| Citric acid | 0.006 g L^−1^ |  | Vitamin B_12_ | 0.1 μg L^−1^ |
| Ferric ammonium citrate | 0.006 g L^−1^ |  | Biotin | 0.1 μg L^−1^ |
| EDTANa_2_ | 0.001 g L^−1^ |  | Thiamine HCl | 10 μg L^−1^ |
| Na_2_CO_3_ | 0.02 g L^−1^ |  | PIV | 6 ml L^−1^ |
| A5 (Trace mental solution) | 1 ml L^−1^ |  | HEPES | 0.5 g L^−1^ |
|  |  |  | Na_2_SiO_3_·9H_2_O | 0.1 g L^−1^ |
|  |  |  | Soil extract | 30 ml L^−1^ |

**Fig. S1.** Overview of material preparation procedures

**Fig. S2.** Fitting a logistic growth curve by estimating intrinsic growth rate (*r*) and carrying capacity (*K*).

**Table S2**. Evaluation of MDA content and activities of SOD, POD, and CAT.

| Sample preparation | 1. Count algal cell 2. Centrifuge a 10 mL sample at 5000 rpm for 20 min 3. Wash by 0.9% sodium chloride solution at 4℃ 4. Ultrasonic crush of algal cells in ice bath ((crush 3s + interval 10s) × 30) 5. Centrifuge at 5000 rpm for 5 min at 4°C, and collect the supernatant 6. Add reagents in sample according to the instructions 7. Preheat spectrophotometer for 30 minutes, adjust to zero with distilled water | | |
| --- | --- | --- | --- |
|  | Absorbance measurement by spectrometer | Calculation formula | Unit |
| MDA content | 532 and 600 nm  *Δ*A = A_532_ − A_600_ | $\frac{25.8\times\Delta A\times V}{n}$ | nmol cells^−1^ |
| SOD activity | 560 nm  IR (inhibition rate) = (A_control_ – A_sample_)/A_control_ | $\frac{11.4\times V\times\frac{\mathrm{IR}}{1-\mathrm{IR}}}{n}$ | U cells^−1^ |
| POD activity | 470 nm  *Δ*A = A_470_ (at 1 min) – A_470_ (at 2 min) | $\frac{2000\times\Delta A\times V}{n}$ | U cells^−1^ |
| CAT activity | 240 nm  *Δ*A = A_240_ (initial) – A_240_ (at 1 min) | $\frac{678\times\Delta A\times V}{n}$ | nmol min^−1^ cells^−1^ |

*n*: number of algal cells in the sample

*V*: volume of sample (mL)
